# Skene's Gland Abscess: A Case Report and Narrative Review

**DOI:** 10.7759/cureus.99383

**Published:** 2025-12-16

**Authors:** Takamasa Tateno, Kohei Urago, Jongmyung Park, Takuya Shimomura, Atsumu Terada

**Affiliations:** 1 Obstetrics and Gynecology, St. Mary’s Hospital, Kurume, JPN

**Keywords:** mri diagnosis, paraurethral cyst, skene’s gland, surgical excision, urethral diverticulum

## Abstract

Skene’s gland cysts and abscesses are uncommon periurethral lesions that can cause chronic vulvar pain and lower urinary tract symptoms but are often underrecognized because their manifestations overlap with more common urological and gynecological conditions. Owing to their close anatomical relationship with the urethra, accurate preoperative diagnosis is essential to distinguish these lesions from urethral diverticulum and avoid inappropriate treatment or urethral injury.

We report the case of a 25-year-old woman with a 12-month history of chronic vulvar pain and urinary symptoms. Physical examination and magnetic resonance imaging (MRI) revealed a cystic lesion lateral to the distal urethra, with T1-hyperintense and T2-hypointense signal characteristics consistent with a Skene’s gland abscess containing viscous purulent material. Under general anesthesia, the cyst wall was completely excised, and purulent material was drained. Intraoperatively, 20 mg of indigo carmine was administered intravenously, and the absence of dye leakage into the operative field confirmed that there was no communication between the cyst and the urethral lumen, allowing safe dissection adjacent to the urethra. The postoperative course was uneventful, and the patient’s symptoms resolved completely.

In addition to this case, we present a narrative review of recently reported Skene’s gland cysts and abscesses in adults and neonates, focusing on the diagnostic contribution of MRI and comparing outcomes of conservative management, marsupialization, and complete excision. This case illustrates how a detailed MRI assessment, combined with intraoperative confirmation of urethral anatomy using indigo carmine, can safely guide complete excision of a Skene’s gland abscess in a symptomatic adult patient. Together with our narrative review, it highlights the need to consider Skene’s gland pathology, alongside urethral diverticulum, in the differential diagnosis of chronic vulvar pain with voiding symptoms and to tailor management according to symptom burden and lesion characteristics.

## Introduction

Skene’s glands, also known as periurethral or paraurethral glands, are paired structures located bilaterally adjacent to the urethral meatus. They contribute to the lubrication of the distal urethra and help prevent urinary tract infections (UTIs) through the secretion of antimicrobial substances. Obstruction of their ducts may lead to the retention of secretions and subsequent cyst formation [1]. Although many Skene’s gland cysts remain asymptomatic and are detected incidentally, enlargement can cause dyspareunia, periurethral discomfort, or voiding difficulties. Secondary infection may result in abscess formation, presenting with acute pain, swelling, and erythema, and may be associated with recurrent UTIs [2].

The true incidence of Skene’s gland cysts is unclear. They are rare in neonates, with an estimated prevalence of 1 per 1,000 to 7,000 births, and are even less frequently reported in adults, suggesting underdiagnosis in clinical practice [3]. Symptoms such as voiding difficulties, urinary frequency, and dyspareunia are nonspecific and overlap with those of other urological or gynecological disorders, which can delay diagnosis. Because of the close anatomical relationship between Skene’s glands and the urethra, cystic lesions in this region must be carefully distinguished from urethral diverticulum and other periurethral masses. Misdiagnosis may lead to inappropriate or incomplete treatment, persistent symptoms, or iatrogenic urethral injury.

Physical examination alone is often insufficient to define the exact origin and extent of periurethral cystic lesions. Magnetic resonance imaging (MRI) plays a central role in clarifying lesion location, internal characteristics (including hemorrhagic or purulent contents), and the presence or absence of communication with the urethral lumen. However, despite the increasing use of pelvic MRI, the literature on Skene’s gland abscess in adults remains limited to small series and isolated case reports, many of which provide only brief descriptions of imaging findings, intraoperative assessment of urethral communication, and long-term outcomes after complete excision. Detailed reports that correlate MRI features with surgical decision-making and postoperative course are relatively scarce.

Here, we describe the case of a young woman with a symptomatic Skene’s gland abscess presenting as chronic vulvar pain and urinary stream disturbance. We focus on the MRI features, intraoperative confirmation of urethral anatomy using indigo carmine to exclude urethral diverticulum, and the rationale for choosing complete cyst wall excision as definitive treatment. In addition, we present a narrative review of recently reported Skene’s gland cysts and abscesses in adults and neonates, emphasizing the diagnostic value of MRI and comparing the outcomes of conservative and surgical approaches. Through this combined case report and narrative review, we aim to raise clinical awareness of this uncommon but clinically relevant entity and provide practical guidance for the diagnosis and management of periurethral cystic lesions.

## Case presentation

A 25-year-old woman presented with a 12-month history of chronic vulvar pain and an abnormal urinary stream. The pain was localized to the left periurethral region and was exacerbated by voiding and prolonged sitting. She also reported deviation and intermittent weakening of the urinary stream but denied hematuria, fever, or other systemic symptoms. Her medical history was unremarkable. At the initial urology visit, a mildly tender cystic lesion was noted near the external urethral orifice. After the initial evaluation, the patient was referred to the gynecology department for further diagnostic workup and treatment planning.

Physical examination and investigations

Pelvic examination revealed a soft, cystic mass on the left side of the external urethral meatus adjacent to the distal urethra. The lesion was mildly tender; however, the overlying mucosa was intact without erythema or ulceration, and no pus or urinary discharge was observed upon compression. Urinalysis revealed no pyuria, hematuria, or bacteriuria, making an active urinary tract infection (UTI) unlikely.

**Figure 1:**
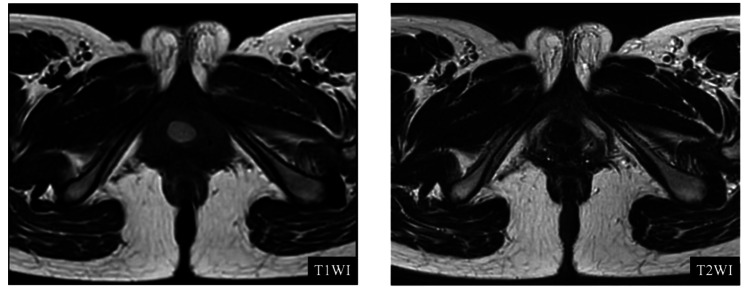
MRI findings (T1- and T2-weighted images) Axial images showed a well-defined cystic lesion lateral to the distal urethra. The lesion demonstrated high signal intensity on T1-weighted imaging and low signal intensity on T2-weighted imaging, with a thin low-signal rim delineating the cyst wall. No communication with the urethra or solid components was observed.

Imaging studies were performed for further evaluation. Transvaginal ultrasonography showed a well-defined cystic lesion, approximately 2 cm in diameter, adjacent to the urethra, with homogeneous internal echogenicity. Additionally, non-contrast pelvic magnetic resonance imaging (MRI) revealed a well-circumscribed lesion with high signal intensity on T1-weighted imaging and low signal intensity on T2-weighted imaging, suggesting a Skene’s gland cyst with hemorrhagic or highly viscous contents (Figure 1). The cyst was located lateral to the distal urethra, and no obvious communication with the urethral lumen or solid components was observed. Based on the clinical presentation and imaging findings, a diagnosis of Skene’s gland abscess was made.

Treatment and clinical course

**Figure 2:**
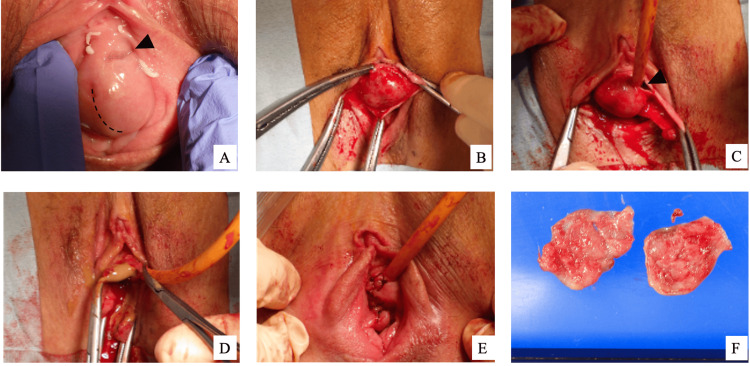
Intraoperative findings (A) Cystic lesion adjacent to the urethra (▶︎). The incision was made along a dotted line away from the urethra. (B) Circumferential dissection of the mucosa surrounding the cyst. (C) Proximity to the urethra confirmed with urethral catheter placement. (D) The cyst was intentionally opened to minimize the risk of urethral injury, and purulent material was drained. The cyst wall was divided and completely excised. (E) Closure with interrupted sutures. (F) Resected specimen.

Considering the patient’s chronic symptoms and imaging findings, complete excision of the cyst was deemed the definitive treatment. The cyst wall was completely excised under general anesthesia. Although preoperative MRI showed no communication with the urethra, 20 mg indigo carmine was intravenously administered at the start of the surgery to reassess potential communication. A catheter was inserted into the urethral meatus, and the mucosa at a site distant from the urethra was incised using a cold knife. The cyst wall was then carefully dissected from the surrounding tissues while continuously confirming its proximity to the urethra (Figures 2A-2C).

The cyst was located near the urethra, and a partial incision allowed visualization of the entire cyst wall from the lumen. The cyst contained dark brown purulent material (Figure 2D). No leakage of indigo carmine was observed, confirming the absence of communication with the urethra. The cyst wall was completely excised while preserving the urethra, and the incision site was closed with interrupted 3-0 Vicryl sutures (Figure 2E).

Postoperatively, the patient’s vulvar pain and voiding difficulties resolved rapidly. Histopathological examination revealed fibrous tissue and chronic inflammatory cell infiltration in the cyst wall, confirming the diagnosis of a Skene’s gland abscess (Figure 2F). The postoperative course was uneventful, and no recurrence has been reported to date. Informed written consent was obtained from the patient for the open-access publication of this case report, including the use of clinical data and images.

## Discussion

Background and epidemiology

The existence of the Skene’s glands was first described in 1672 by the Dutch anatomist, Reinier de Graaf; however, their anatomical details were systematically characterized in 1880 by the Scottish gynecologist Alexander Johnston Chalmers Skene [4].

Skene’s gland cysts are extremely rare and often underrecognized, with relatively few case reports available in the medical literature. They can be broadly classified as congenital or acquired. Congenital cysts occur in approximately 1 in 1,000 to 7,000 births, whereas adult-onset cysts are most commonly reported in women in their 30s and 40s [5]. Despite their rarity, these lesions may present with nonspecific lower urinary tract or vulvar symptoms and may be misdiagnosed as more common conditions, underscoring their clinical relevance and the need for greater diagnostic awareness.

Pathophysiology and clinical manifestations

The pathogenesis of Skene’s gland cysts is primarily attributed to obstruction of the glandular ducts, which leads to the accumulation of secretions and subsequent cyst formation. The obstruction is most commonly caused by bacterial infection, with pathogens such as Neisseria gonorrhoeae, Escherichia coli, and other vaginal flora frequently implicated [4]. Other proposed mechanisms, including cystic degeneration of embryological remnants and hormonal influences, have been suggested but remain hypothetical [5].

Clinically, most Skene’s gland cysts are small, typically less than 1 cm in diameter, and asymptomatic, often detected incidentally during routine pelvic examinations. When the cyst enlarges, it may cause dyspareunia, dysuria, periurethral tenderness, or local discomfort. In some cases, compression of the urethra can impair urinary flow, resulting in a weak or interrupted stream and, rarely, acute urinary retention [5]. The chronic vulvar pain and urinary stream disturbance observed in the present case are consistent with such a mass effect on the distal urethra.

Infection of the Skene’s glands can sometimes be mistaken for a UTI, as routine urinalysis may not detect the responsible organisms; bacteria confined within the glands may not be sufficiently shed into the urine, making accurate diagnosis challenging. Although antibiotic therapy may provide transient relief, persistent bacterial colonies within the glands can lead to recurrent symptoms. This recurrent pattern highlights the importance of considering Skene’s gland disease in women presenting with chronic or recurrent UTI-like symptoms [6].

Diagnostic methods

When symptomatic, a Skene’s gland cyst may present as a mobile, cystic mass near the distal urethra [7]. Although physical examination is crucial, differentiating it from other periurethral cystic lesions, particularly urethral diverticulum, can be challenging [5]. Therefore, imaging plays a key role in establishing a definitive diagnosis.

Ultrasonography is a noninvasive and readily available modality that can confirm the cystic nature of the lesion [6]. When differentiation from other periurethral pathologies remains uncertain, MRI provides superior diagnostic accuracy by delineating the cyst’s exact location, morphology, internal composition, such as purulent or hemorrhagic content, and potential communication with the urethra. On T2-weighted images, Skene’s gland cysts typically appear as well-defined, hyperintense lesions located lateral to the urethra and inferior to the pubic symphysis [7]. The presence of wall thickening or heterogeneous internal signal intensity suggests infection or inflammation, consistent with the findings in the present case. In our patient, the lateral periurethral location of the lesion, the characteristic T1-hyperintense/T2-hypointense signal pattern, and the absence of a demonstrable neck or visible communication with the urethral lumen on MRI supported the diagnosis of a Skene’s gland abscess rather than a urethral diverticulum.

Differential diagnosis

Skene’s gland cysts must be distinguished from other periurethral or vulvar cystic lesions that may present with similar clinical features. The main differential diagnoses, including urethral diverticulum, Gartner duct cyst, and urethral caruncle, are summarized in Table 1. Among these, urethral diverticulum is the most important differential diagnosis because of its close anatomical relationship to the urethra and overlapping symptoms, which can make the distinction challenging. MRI is particularly valuable for determining whether the lesion communicates with the urethral lumen and for confirming the diagnosis [8]. As summarized in Table 1, a urethral diverticulum typically demonstrates a definite neck communicating with the urethral lumen, whereas Skene’s gland cysts and abscesses are located lateral to the distal urethra without visible communication. When additional anatomical detail is required, retrograde or voiding cystourethrography can be performed to further evaluate the presence and extent of a diverticulum and its connection to the urethral lumen [9].

**Table 1:** Comparison of major differential diagnoses in terms of pathology, imaging, and treatment

Lesion	Typical Location / Age Group	Communication with Urethral Lumen	Key MRI Findings	Typical Treatment
Skene’s gland cyst or abscess	Posterolateral to distal urethra / Adults, Neonates	No	T1: low / T2: high (T1 ↑ / T2 ↓ with infection or hemorrhage)	Antibiotics ± drainage, excision or marsupialization [10]
Urethral diverticulum (UD)	Mid- to distal urethra / Adults	Yes	Visualization of diverticular neck	Diverticulectomy with reconstruction [11]
Gartner duct cyst	Anterolateral vaginal wall / Adolescents–Adults	No	Cyst located away from the urethra	Observation or excision [12]
Urethral caruncle	Posterior urethral meatus / Postmenopausal women	No	T1: low / T2: high	Observation or excision [13]

Treatment strategies

The management of Skene’s gland cysts depends on symptom severity and underlying pathology. Asymptomatic cases generally require no intervention and can be managed conservatively with observation [5]. When symptoms such as pain, voiding difficulties, or recurrent infections develop, especially in the setting of documented infection or significant mass effect on the urethra, surgical treatment becomes necessary [8,10].

Various procedures have been described, including aspiration, marsupialization, and complete excision [1]. Although aspiration is simple, it carries a high risk of recurrence, whereas marsupialization provides continuous drainage and reduces this risk [10,11]. Complete excision of the cyst wall, however, offers the most definitive and curative approach, particularly in cases complicated by chronic infection or recurrent inflammation [12,13]. In symptomatic adult patients, several reports have shown favorable outcomes and low recurrence rates after complete excision, whereas more conservative approaches are often reserved for minimally symptomatic or neonatal cases. In the present case, total excision was selected as the optimal treatment based on the patient’s chronic vulvar pain, urinary stream disturbance, and MRI findings consistent with an infected Skene’s gland cyst. This strategy resulted in complete symptom resolution and no recurrence, thereby underscoring the efficacy and safety of complete cyst wall excision in appropriately selected patients.

Literature review

Table 2 summarizes the clinical characteristics, imaging findings, and treatment outcomes of previously reported cases in comparison with the present case. As shown in Table 2, previous studies consistently emphasize the importance of accurate imaging and complete surgical excision in managing Skene’s gland cysts and abscesses, particularly in symptomatic adult patients, whereas conservative management is often sufficient in neonatal cases. Shah et al. (2012) reported that most patients experienced symptom improvement after surgical removal, while Foster et al. (2016) observed no recurrence following complete excision [14,15]. Mahfouz et al. and Tamburrini et al. highlighted the diagnostic value of MRI, particularly for non-palpable or complex lesions, and its role in differentiating Skene’s gland pathology from urethral diverticulum and other periurethral masses [7,16]. Neto et al. (2023) described three neonatal cases that resolved spontaneously without surgical intervention, supporting the role of conservative management in selected patients with small, asymptomatic, or minimally symptomatic lesions [17]. Although simple aspiration has been associated with a high recurrence rate, complete cyst wall excision remains the most reliable curative approach in adults, whereas observation or minimal drainage is often sufficient in neonates [17,18]. In the present case, preoperative MRI revealed no urethral communication, which was confirmed intraoperatively using indigo carmine before circumferential excision to achieve curative resection. These findings are consistent with the adult literature and illustrate how detailed imaging combined with intraoperative assessment can guide the choice of definitive surgical management.

**Table 2:** Comparison of the present case with major published reports

	Case	Key Findings	Treatment	Outcome / Recurrence
Present case (2025)	25 years old	Cyst lateral to the urethra on MRI Cyst wall excision with pus drainage	Complete excision	Good postoperative course; no recurrence at 6 months
Shah et al., 2012 [14]	34 cases	MRI with urethroscopy or cystoscopy	Surgical excision	88.2% initial response; 85.3% overall symptom improvement
Foster et al., 2016 [15]	10 cases	Diverticulum excluded by MRI Cystoscopy prior to excision	Complete excision	No recurrence during long-term follow-up
Mahfouz et al., 2022 [7]	57 cases	Urethral diverticulum (64.9%), Skene’s cyst (14%) MRI	Treatment based on pathology	No recurrence over a mean 35-month follow-up
Tamburrini et al., 2021 [16]	48 years old	Non-palpable case MRI confirmed the diagnosis	Antibiotics, marsupialization	Symptom resolution
Tzelepis et al., 2023 [19]	67 years old	Large cyst; MRI endoscopy for diagnosis	Incision, drainage + marsupialization	Good outcome at 24 months
Karadeniz et al., 2024 [5]	Adult	Resembled cystocele but differentiated by MRI	Complete excision	Good outcome
Shin et al., 2021 [3]	34 years old	Initial aspiration with recurrence, then excision	Complete excision	No recurrence at 1 year
Moralioğlu et al., 2013 [18]	Neonate	Urethral meatus deviation; milky contents	Incision, drainage	No recurrence at 9 months
Neto et al., 2023 [17]	3 neonates	Spontaneous resolution reported	Observation	Spontaneous resolution

## Conclusions

Skene’s gland cysts are uncommon and often underrecognized periurethral lesions that can mimic other pathologies, such as urethral diverticulum, leading to delayed diagnosis and inappropriate management. In our patient with chronic vulvar pain and urinary stream disturbance, a combination of careful physical examination and detailed MRI, demonstrating a lateral periurethral cystic lesion without visible communication to the urethral lumen, guided the diagnosis of a Skene’s gland abscess. Intraoperative indigo carmine testing further confirmed the absence of urethral communication and allowed the safe, complete excision of the cyst wall immediately adjacent to the urethra while preserving urinary function.

Based on this case and the limited number of published reports summarized in our narrative review, conservative management may be appropriate for asymptomatic lesions and for many neonatal cases, in which spontaneous resolution or improvement has been described. In contrast, surgical intervention, particularly complete cyst wall excision, appears to be the most reliable curative option for symptomatic or infected Skene’s gland lesions in adults, with low recurrence rates when urethral anatomy is carefully assessed preoperatively and intraoperatively. This case underscores the importance of early recognition of Skene’s gland pathology in the differential diagnosis of chronic vulvar pain with voiding symptoms, comprehensive imaging, and operative evaluation to exclude urethral diverticulum, and individualized treatment planning tailored to lesion characteristics and patient symptoms in order to achieve favorable outcomes and prevent recurrence.
